# Dynamic transcriptome and metabolome analyses of two types of rice during the seed germination and young seedling growth stages

**DOI:** 10.1186/s12864-020-07024-9

**Published:** 2020-08-31

**Authors:** Jing Yang, Ling Su, Dandan Li, Lixin Luo, Kai Sun, Meng Yang, Fengwei Gu, Aoyun Xia, Yongzhu Liu, Hui Wang, Zhiqiang Chen, Tao Guo

**Affiliations:** National Engineering Research Center of Plant Space Breeding, South China Agricultural University, Guangzhou, 510642 China

**Keywords:** Rice, Germination, Seedling growth, Transcriptome, Metabolome

## Abstract

**Background:**

Seed germination and young seedling growth are important agricultural traits for developing populations of both irrigated and directly seeded rice. Previous studies have focused on the identification of QTLs. However, there are few studies on the metabolome or transcriptome of germination and young seedling growth in rice.

**Results:**

Here, an *indica* rice and a *japonica* rice were used as materials, and the transcripts and metabolites were detected during the germination and young seedling growth periods on a large scale by using RNA sequencing and a widely targeted metabolomics method, respectively. Fourteen shared transcripts and 15 shared metabolites that were continuously differentially expressed in the two materials were identified and may be essential for seed germination and young seedling growth. Enrichment analysis of differentially expressed genes in transcriptome expression profiles at different stages indicated that cell wall metabolism, lipid metabolism, nucleotide degradation, amino acid, etc., were enriched at 0–2 days, and most of the results are consistent with those of previous reports. Specifically, phenylpropanoid biosynthesis and glutathione metabolism were continuously enriched during the seed germination and young seedling growth stages. Next, KO enrichment analysis was conducted by using the differentially expressed genes of the two materials at 2, 3 and 4 days. Fourteen pathways were enriched. Additionally, 44 differentially expressed metabolites at 2, 3 and 4 days were identified. These metabolites may be responsible for the differences in germination and young seedling growth between the two materials. Further attention was focused on the ascorbate–glutathione pathway, and it was found that differences in ROS-scavenging abilities mediated by some APX, GPX and GST genes may be directly involved in mediating differences in the germination and young seedling growth speed of the two materials.

**Conclusions:**

In summary, these results may enhance the understanding of the overall mechanism of seed germination and young seedling growth, and the outcome of this study is expected to facilitate rice breeding for direct seeding.

## Background

Rice (*Oryza sativa*) is one of the most important crops worldwide and is the principal food of nearly 50% of the world population [1]. Recently, direct-seeded rice (DSR) has become increasingly popular worldwide because of its cost efficiency and convenience. Uniform, rapid germination and vigorous seedling growth are essential for the direct seeding of rice [2]. Therefore, further exploration of the genetic and molecular mechanisms controlling seed germination and seedling growth is an important objective of direct-seeded rice breeding.

Seed germination is a quantitative trait controlled by multiple genes and environmental factors during seed germination stage. To date, cloned genes related to the control of rice seed germination are still limited. The first gene related to rice seed germination that was cloned was *qLTG-3-1*, which controls the germination rate (GR) under various conditions; this gene encodes a protein with an unknown function [3]. The second rice seed germination-related gene that was cloned was *OsTPP7*, which controls anaerobic germination and can enhance anaerobic germination tolerance [4]. The third rice seed germination-related gene that was cloned was *OsSAP16,* which encodes a zinc-finger domain protein as a major causal gene for low-temperature germination [5]. Recently, He et al. (2018) reported an isopropylmalate synthase gene and that the disruption of *OsIPMS1* resulted in low seed vigor under various conditions [6]. The progress that has been made for seedling growth is relatively little compared to that for germination. One major quantitative trait locus (QTL) for seedling height (SH) on the long arm of chromosome 3 has been finely mapped, and *OsGA20ox1* is the most likely candidate at the locus [7, 8].

Germination is a series of events that begins with imbibition, during which dry seeds uptake water, followed by the reinitiation of metabolic processes and the elongation of the embryonic axis [9]. Seed germination and subsequent young seedling growth require a large number of energies and nutrients, however, due to lack of the mineral uptake system and photosynthetic apparatus in germinated seeds, so, these energies and nutrients can only be obtained from seed reserves [9]. There are three respiratory pathways, the glycolysis, the pentose phosphate pathway, and the tricarboxylic acid (TCA) cycle, are activated in the imbibed seeds when dry seeds imbibe water [10]. And the glycolysis and the TCA cycle provide most of the energy needed for seed germination.

In addition to the intensive changes in both gene expression and metabolic activities during seed germination, the levels of many signaling molecules, such as nitric oxide (NO) and reactive oxygen species (ROS), also change drastically [11–13]. Furthermore, gibberellins (GAs) and abscisic acid (ABA) are two major plant hormones that control seed dormancy and germination [14, 15]. GA promotes seeds germination, whereas, ABA promotes the induction and maintenance of seed dormancy. It has been suggested that germination is regulated by the antagonistic effects of ABA and GA. During the germination stage, ROS are continuously produced, and provided that the balance between production and scavenging tightly regulates their accumulation, these toxic molecules now appear to be beneficial to germination [16]. In fact, hydrogen peroxide (H_2_O_2_) [17], NO [18] and superoxide radicals (O_2_^.−^) [19, 20] have been shown to accumulate during seed germination in various species. However, the mechanism of the ROS-scavenging system during seed imbibition or germination has not been widely reported.

The expansion of high-quality sequence data and the establishment of large-scale omics technologies, in particular, metabolomics, can effectively help us analyze the complex process of rice seed germination and seedling growth as a whole. Metabolites are the end products of cellular processes and represent the ultimate reflection of the response of biological systems to genetic or environmental changes [21]. Rice seed germination has been studied for several decades; however, there are few studies on the metabolome or transcriptome of germination in rice. Shu et al. (2008) used a metabolite profiling approach based on gas chromatography−mass spectrometry (GC − MS) to investigate time-dependent metabolic changes in the course of the germination of rice. Brown rice kernels were soaked and incubated, and samples were taken after 0, 24, 48, 72, and 96 h. Investigation of the obtained fractions by GC resulted in the detection of 615 distinct peaks, of which 174 were identified by means of MS [22]. Transcriptome and metabolite profiling of rice (*Oryza sativa* L.) embryo tissue were carried out, sampling transcripts at 0 and 1, 3, 12, and 24 h and metabolites at 0, 1, 3, 6, 12, 24, and 48 h after imbibition, resulting in a final set of 24,150 detected transcripts and 126 detected metabolites. A detailed analysis revealed that 1 hour after imbibition, rapid changes in metabolism occurred, which independent of changes in transcript levels. However, later changes in the metabolome, such as carbohydrate, amino acid, and cell wall metabolism, seemed to be driven by increases in transcript levels [23].

Summarizing previous omics studies, we find that, first, the amount of metabolites detected is small; second, the sampling time of transcriptome analysis is typically 24 h, and thus, later changes, especially in the seedling stage and potentially regulatory changes in the transcriptome, have not yet been thoroughly investigated in rice; and third, the embryo rather than the whole plant has been studied. Here, the *indica* rice variety YZX and *japonica* rice variety 02428 were used as materials. All tissues were sampled at 0, 2, 3, and 4 days after imbibition. The transcripts and metabolites were detected on a large scale by using RNA sequencing (RNA-Seq) and a widely targeted metabolomics method, respectively. These findings enable us to reveal the regulatory mechanism of rice seed germination and young seedling growth from the level of transcription and metabolism. Moreover, we further investigated the differences in the transcripts and metabolites of the two materials during germination and young seedling growth and revealed the regulatory mechanism of the differences in the growth rates of the two materials during germination and young seedling growth.

## Results

### Differences in germination and young seedling growth abilities between 02428 and YZX

**Fig. 1:**
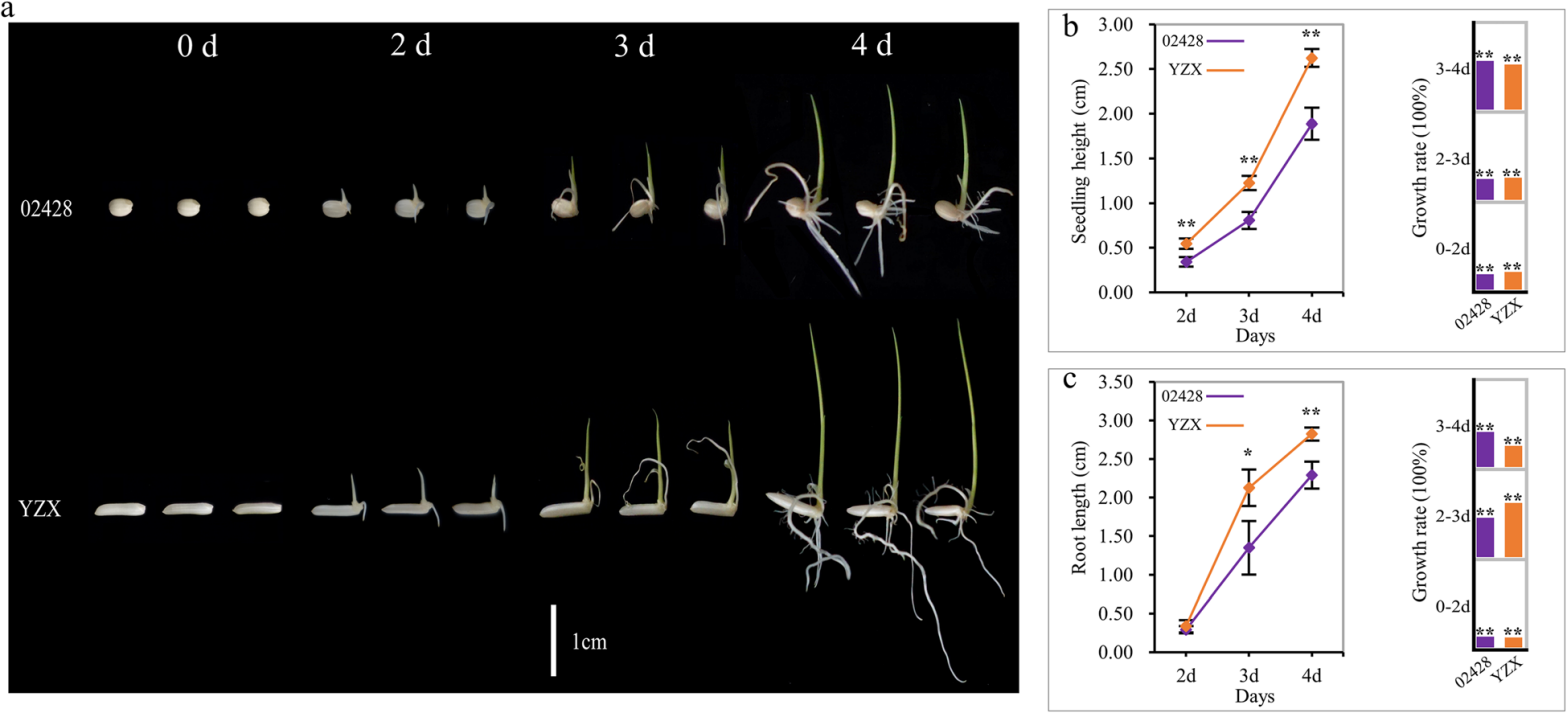
Germination and young seedling growth characteristic of 02428 and YZX. **a** Phenotypes of germination and growth of 02428 and YZX from 0 to 4 days. **b** Changes in seedling height of 02428 and YZX from 0 to 4 days. **c** Changes in root length of 02428 and YZX from 0 to 4 days

From the phenotypes shown in Fig. 1, it is clear that the speed of germination and young seedling growth of YZX were significantly faster than those of 02428. In particular, the seedling height of YZX was significantly higher than that of 02428 at 2, 3, and 4 days. The root length of YZX was significantly higher than that of 02428 at 3 and 4 days. Furthermore, we calculated the growth rate of the seedling height and root length of the two materials. Both the aboveground and belowground of the two materials grew very quickly from 0 to 4 days, and the growth rate was highly significant between days, from the 3rd to the 4th day, with the largest change in the growth of seedling height, and from 2nd to the 3rd day, with the largest change in the growth of root length.

### Identification of the core genes and metabolites that respond to rice germination and young seedling growth

**Fig. 2:**
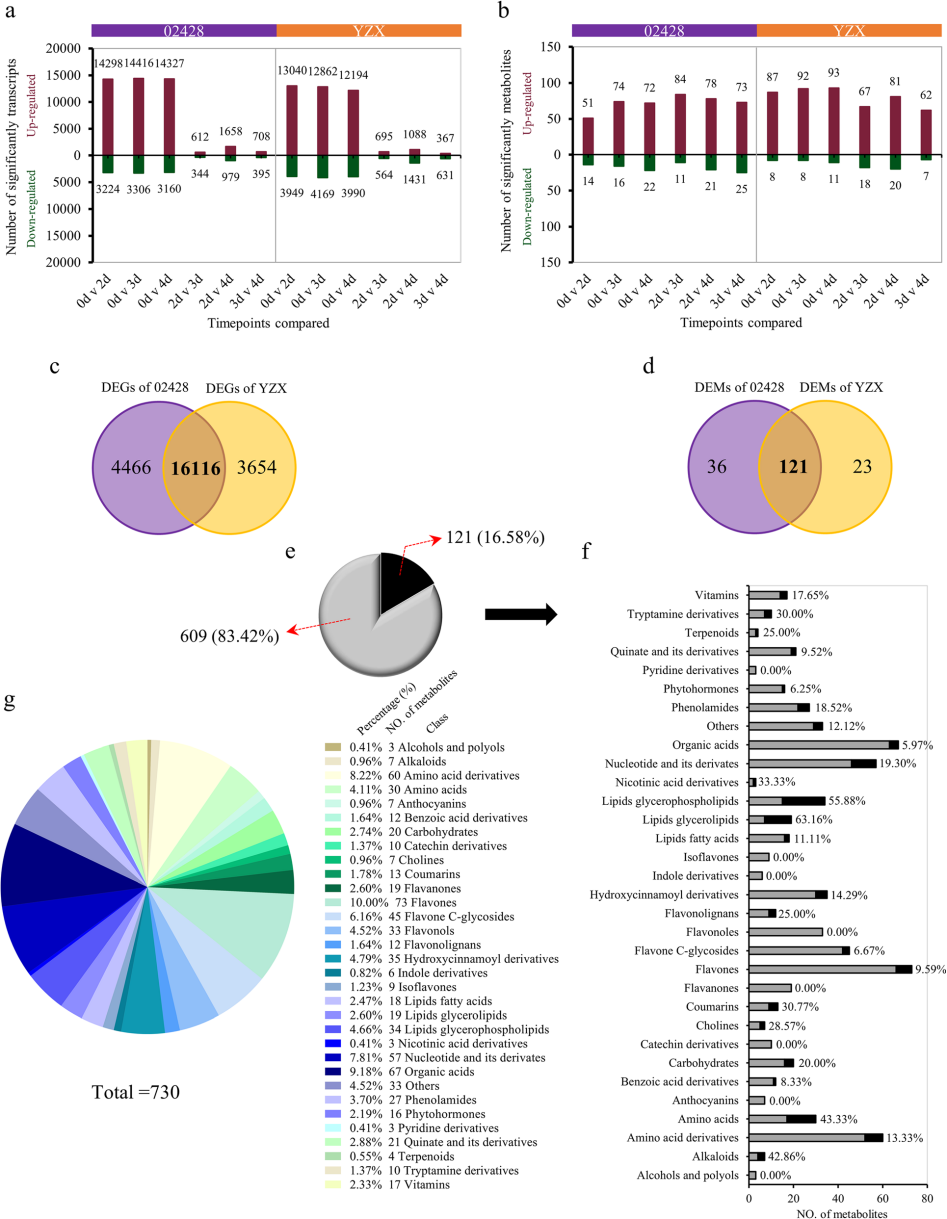
Summary of changes in transcript and metabolite abundance in rice in response to germination and young seedling growth. **a**, **b** Summary of the number of significant changes in transcripts and metabolites between different time points. **c**, **d** Venn diagram of DEGs and DEMs between 02428 and YZX. **e** A total of 121 core metabolites accounted for the proportion of total metabolites. **f** The number and proportion of 121 core metabolites in various metabolites and their derivatives are detailed, and the black box represents the core metabolites. **g** Classification of 730 metabolites

To fully understand the transcriptional and metabolic changes in rice seed germination and young seedling growth, a widely targeted metabolomics and RNA-Seq analysis was performed on the whole germinating seeds, including the seed and seedling, on days 0, 2, 3, and 4. For RNA-Seq, three independent replicates per time point and a total of 24 libraries were sequenced. For each sample, 25,087,077.33 high-quality clean reads were generated on average, of which an average of 20,207,241.88 reads could be mapped to the transcripts annotated in the *japonica* Nipponbare reference genome IRGSP-1.0. The average rates of mapped reads for 02428 and YZX samples in the Nipponbare reference genome were 80.94 and 80.27%, respectively (Additional file 1). To identify genes that were differentially expressed during seed germination and young seedling growth, pairwise differential expression profiling analysis (FDR ≤ 0.05, absolute value fold change ≥ 2.0) at all time points was performed. A total of 20,582 and 19,770 differentially expressed genes (DEGs) were identified in 02428 and YZX, respectively (Additional file 2). When successive time points were compared, the majority of DEGs were identified between 0 and 2 days, while the number of DEGs identified in the comparisons of 2 days v 3 days and 3 days v 4 days was considerably reduced. Interestingly, over time, the number of upregulated DEGs was higher than the number of downregulated DEGs (Fig. 2a).

For metabolite profiling analysis, each timepoint was repeated three times, and a total of 24 samples were obtained. Ultra-high-performance liquid chromatography (UPLC, Shim-pack UFLC SHIMADZU CBM30A, http://www.shimadzu.com.cn/) and tandem mass spectrometry (MS/MS, Applied Biosystems 6500 QTRAP, http://www.appliedbiosystems.com.cn/) were used to obtain mass spectrometric data of the 24 samples. Based on the self-established database Metware database (MWDB) and a public database of metabolite information, the samples were qualitatively and quantitatively analyzed by MS. In total, 730 metabolites were identified, including 32 substances and their derivatives (Fig. 2g and Additional file 3). The significant differences in the relative metabolite content was tested by OPLS-DA based on a VI*P* value ≥1 and a univariate statistical analysis of the test value *P* value > 0.05. Similar to the transcriptome, we performed a pairwise analysis of all time points. A total of 157 and 144 differentially expressed metabolites (DEMs) were identified in 02428 and YZX, respectively (Additional file 3). Over time, the number of upregulated DEMs was higher than the number of downregulated DEMs. Notably, different from the transcriptome, the amount of differentially identified metabolites varied little at each period (Fig. 2b).

The varieties 02428 and YZX belong to two subspecies. Therefore, the genes and metabolites that respond to the germination and young seedling growth of these two materials should be different. Notably, there were some overlapping DEGs (16,116) and DEMs (121) in both materials (Fig. 2c, d). We believe that these overlapping DEGs and DEMs are “core DEGs and DEMs” that may be necessary for rice seed germination and young seedling growth.

The 121 core metabolites accounted for 16.58% of the total metabolites, accounting for 24 of 32 kinds of metabolites. The number of lipids glycerolipids, lipids glycerophospholipids and amino acids were found to be relatively higher in the core metabolites, accounting for a higher proportion of all of the different kinds of metabolites. (Fig. 2e, f).

To further understand the functions and pathways of core DEGs during seed germination and young seedling growth. We performed GO and KEGG enrichment analyses for the core DEGs.

An FDR < 0.05 was taken as the threshold in both enrichment analyses. With respect to the cellular component GO term, these core DEGs were significantly enriched in 8 terms, including extracellular region, plastid, and cell periphery. In terms of the biological process GO term, these core DEGs were mainly significantly involved in 12 processes, including single-organism metabolic process, carbohydrate metabolic process and lipid metabolic process. With respect to the molecular function GO term, these core DEGs were mainly related to 12 items, including catalytic activity, transporter activity and receptor activity (Additional file 4: Figure S1a, b, c). KEGG analyses provided a different insight into the molecular understanding of the germination and young seedling growth processes in rice. The results showed that a total of 34 terms were enriched, which mainly included amino acid metabolism, carbohydrate metabolism, energy metabolism, biosynthesis of other secondary metabolites, lipid metabolism and signal transduction (Additional file 4: Figure S1d).

### Identification of continuously differentially expressed genes and metabolites in the two materials

**Fig. 3:**
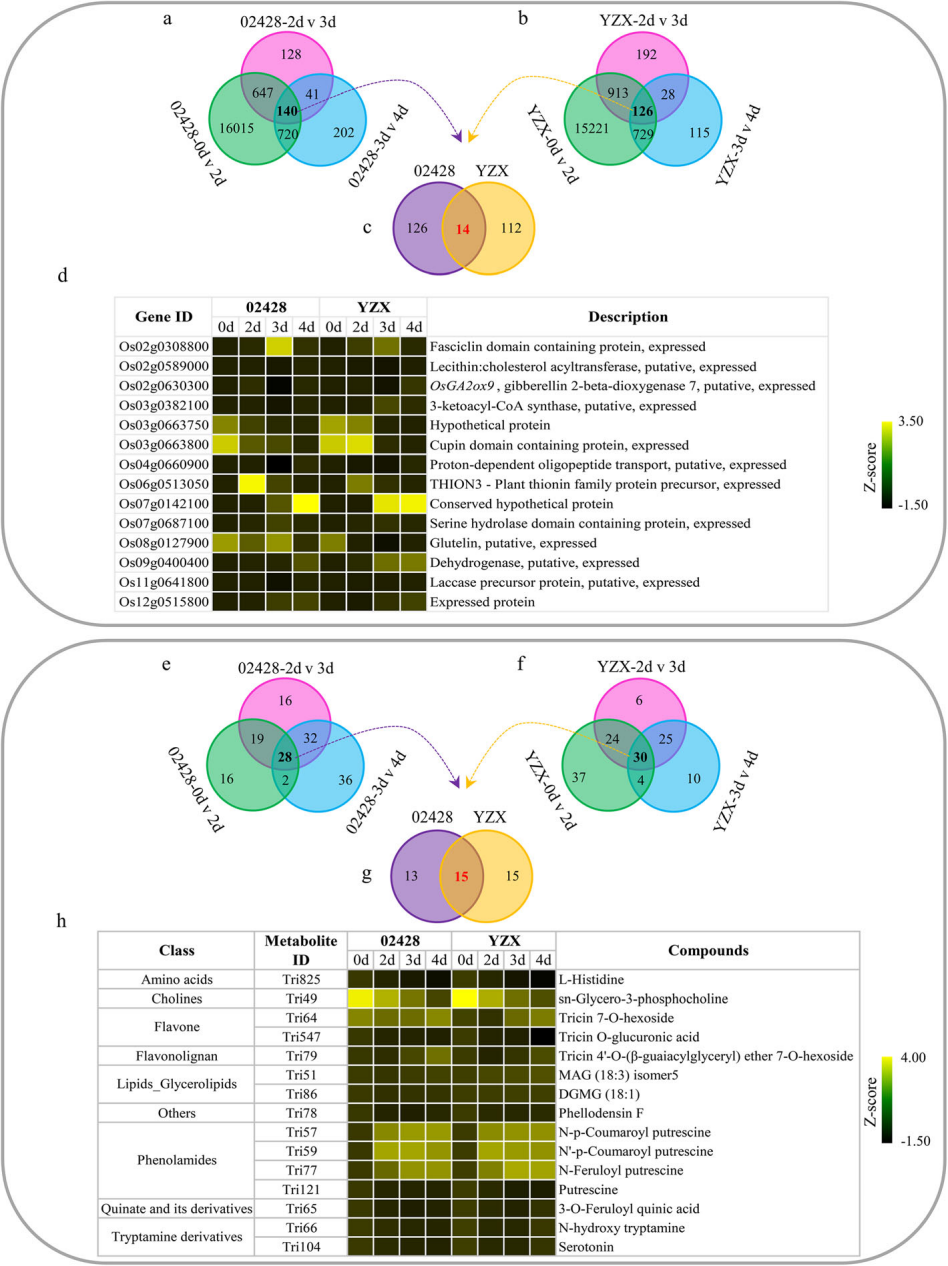
Identification of continuously differentially expressed genes and metabolites. **a** Venn diagram of DEGs during the three periods of 02428. **b** Venn diagram of DEGs during the three periods of YZX. **c** Venn diagram for 02428 and YZX based on continuously DEGs. **d** Heat maps of 14 continuously DEGs. Gene expression was standardized by the z-score method, with the yellow color representing high expression and the black color representing low expression. **e**, **f**, **g** and **h** The analyses of the contents of the corresponding metabolites

We identified core genes and metabolites that responded to germination and young seedling growth, but they were not differentially expressed at all timepoints. Therefore, we also identified genes and metabolites with continuous differential expression in all three periods (0 v 2 days, 2 v 3 days, and 3 v 4 days). There were 140 genes in 02428 and 126 genes in YZX that were continuously differentially expressed. Only 14 genes from the two materials overlapped (Fig. 3a, b, c; Additional file 5). Among them, the expression levels of *cupin domain-containing protein* (Os03g0663800) and *glutelin* (Os08g0127900) were continuously downregulated (Fig. 3d), which is consistent with the reported expression profiles of seed germination [23]. At the same time, we also identified 28 and 30 continuously DEMs in 02428 and YZX, respectively (Fig. 3e, f). The two materials shared 9 classes of 15 metabolites (Fig. 3g; Additional file 5). Among them, phenolamides were the most, with a total of 4, and the relative content of three of them was continuously upregulated (Fig. 3h).

### Enrichment analysis of differentially expressed genes in transcriptome expression profiles at different stages

**Fig. 4:**
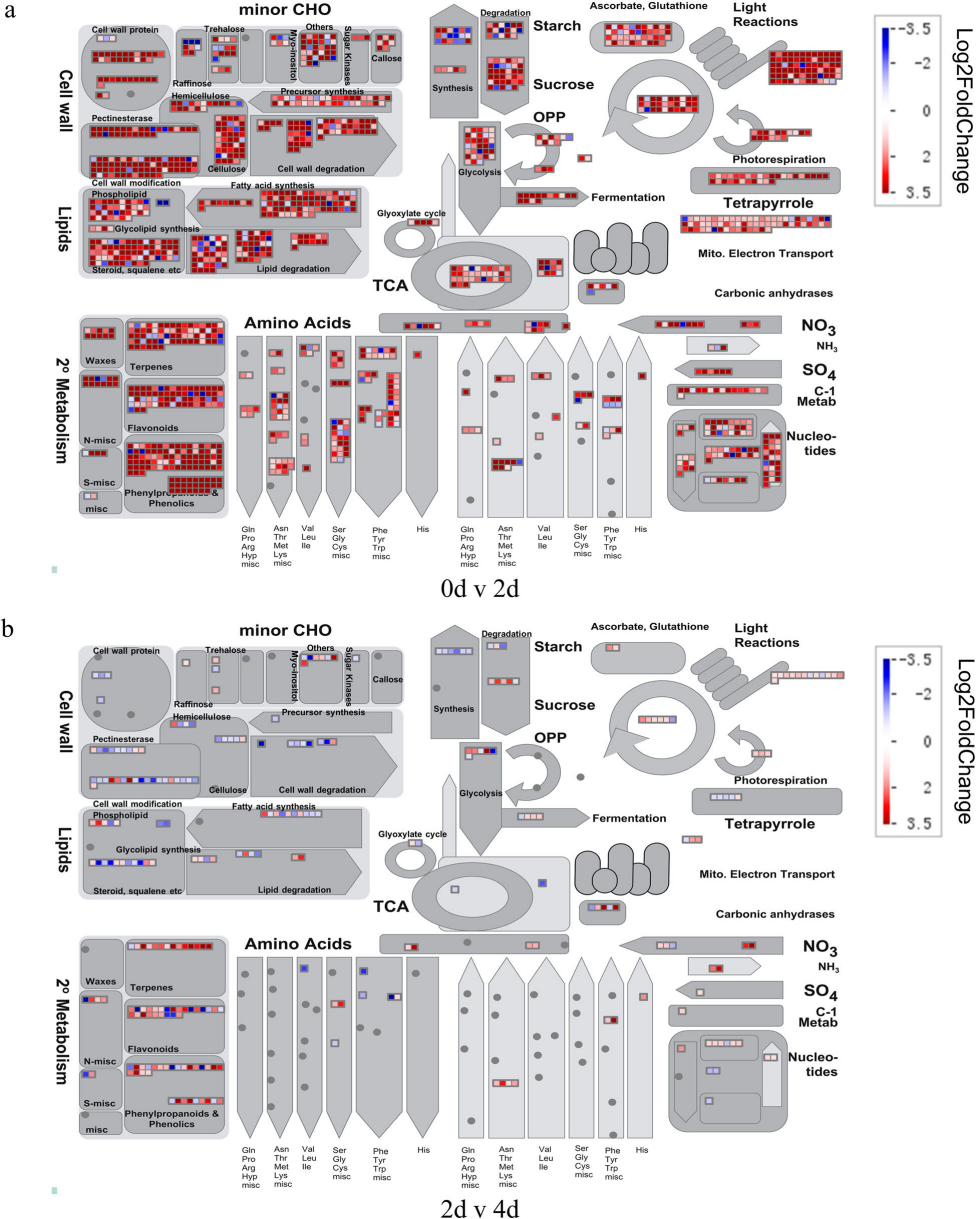
MapMan metabolism overview maps showing differences in transcript levels during seed germination and young seedling growth of 02428. **a** 0 days v 2 days. **b** 2 days v 3 days. Log2 ratios for average transcript abundance were based on three replicates. The log_2_-fold change color scale ranges from − 3.5 to 3.5, where blue represents downregulated transcripts, and red represents upregulated transcripts

Figure 1 shows that the germination of the two materials occurs mainly from 0 to 2 days, while 2–4 days is mainly the growth of the young seedlings. To review the genome-wide transcriptomic changes at the germination and young seedling growth stages, MapMan software [24] was used to identify metabolic pathways and biological processes during the two stages to visualize overall transcriptomic changes. The results show that 02428 and YZX are very similar, i.e., almost all items were upregulated at the germination stage (0–2 days), especially cell wall metabolism, lipid metabolism, secondary metabolism, amino acid metabolism, starch and sucrose metabolism, glycolysis, TCA cycle, and mitochondrial electron transport (Fig. 4a; Additional file 6: Figure S2a). Although there were fewer DEGs in the young seedling growth stage (2–4 days) than in the germination stage, there were DEGs associated with cell wall metabolism, lipid metabolism, and secondary metabolism in both 02428 and YZX. Notably, these DEGs were mostly downregulated in YZX. However, unlike YZX, the DEGs associated with secondary metabolism pathways such as terpenes, flavonoids and phenylpropanoids and phenolics were mostly upregulated in 02428 (Fig. 4b; Additional file 6: Figure S2a).

**Fig. 5:**
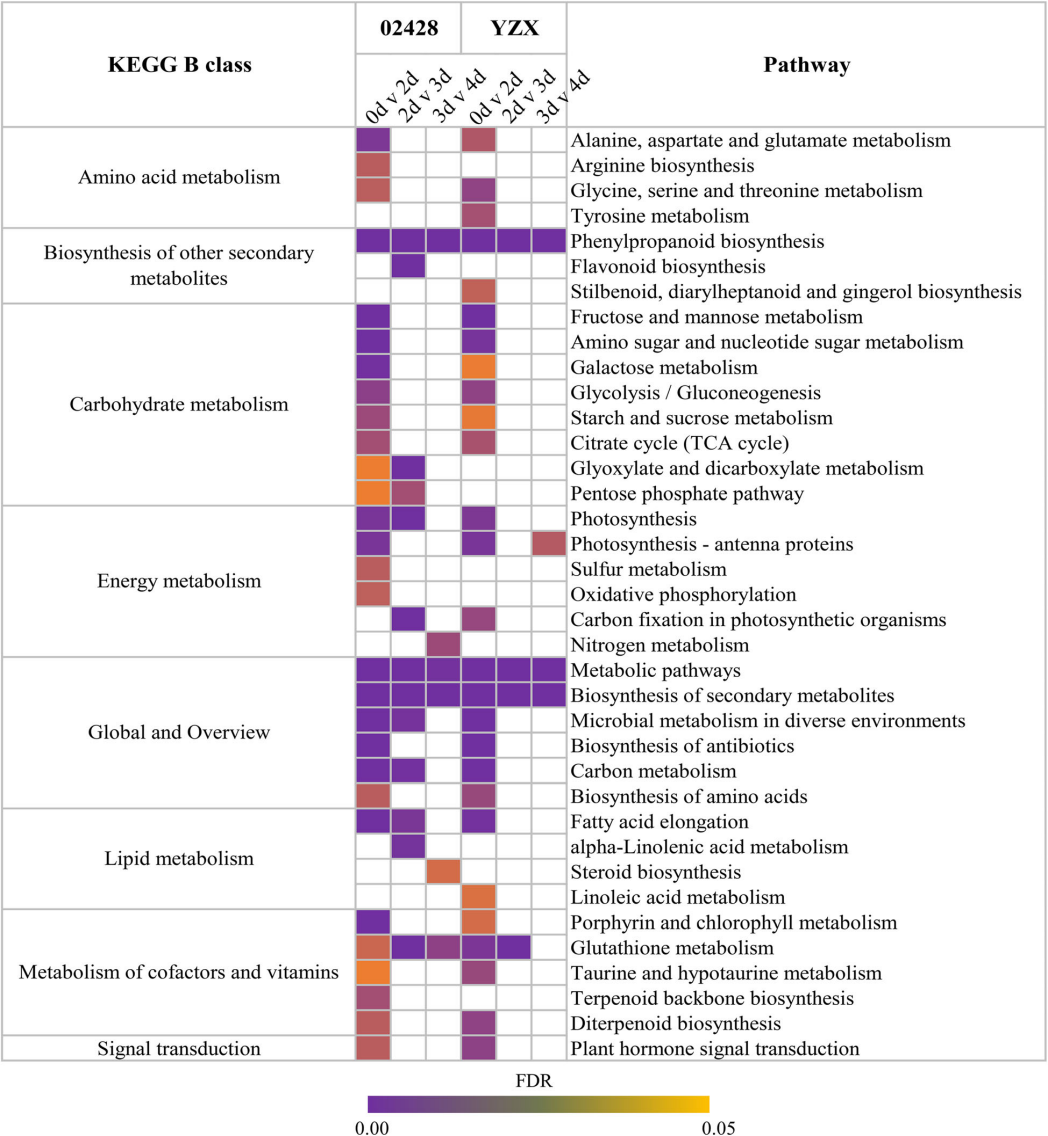
KEGG analyses for the DEGs (0 v 2 days, 2 v 3 days, and 3 v 4 days) in 02428 and YZX. Different colors show the values of FDR, and blanks indicate items that were not significantly enriched during this period

To further clarify the transcriptome changes at different periods, dynamic KEGG Orthology (KO) and GO enrichment analyses of the expression profiles of the two materials were performed. An FDR < 0.05 was taken as the threshold in both enrichment analyses. The results show that the number of enriched items during the germination stage (0–2 days) was much higher than that during the young seedling stage (2–4 days). In terms of the KEGG analysis, the DEGs (0 days v 2 days) of 02428 were mainly significantly involved in 29 pathways, and the DEGs (0 days v 2 days) of YZX were mainly significantly involved in 27 pathways. Notably, there were 23 pathways that were shared by both materials, including carbohydrate metabolism, energy metabolism, metabolism of cofactors and vitamins and amino acid metabolism. The DEGs of 02428 were enriched in 13 pathways during day 2 and day 3, while YZX had only 4 pathways. They shared 4 pathways, including glutathione metabolism and the biosynthesis of secondary metabolites, such as phenylpropanoid biosynthesis. The DEGs of 02428 were enriched in 6 pathways during day 3 and day 4, while YZX had 4 pathways. They shared 3 pathways, including phenylpropanoid biosynthesis in the biosynthesis of secondary metabolites. Interestingly, phenylpropanoid biosynthesis was significantly enriched at three periods in both materials. Surprisingly, in the GO enrichment analysis results, the secondary metabolic process was also significantly enriched at three periods in both materials (Fig. 5; Additional file 7). This suggests that the biosynthesis of secondary metabolites, particularly phenylpropanoid biosynthesis, and glutathione metabolism are involved in seed germination and young seedling growth.

### Glutathione metabolism and phenylpropanoid; flavonoid; and stilbenoid, diarylheptanoid and gingerol biosynthesis may be involved in mediating the differences in the speed of 02428 and YZX germination and young seedling growth

The germination and young seedling growth speeds of 02428 and YZX are quite different. Therefore, we not only focused on the transcription of the two materials in the growth and development process but also explored the reasons for the faster germination and young seedling growth in YZX than in 02428 from the perspectives of the transcriptome and metabolome.

**Fig. 6:**
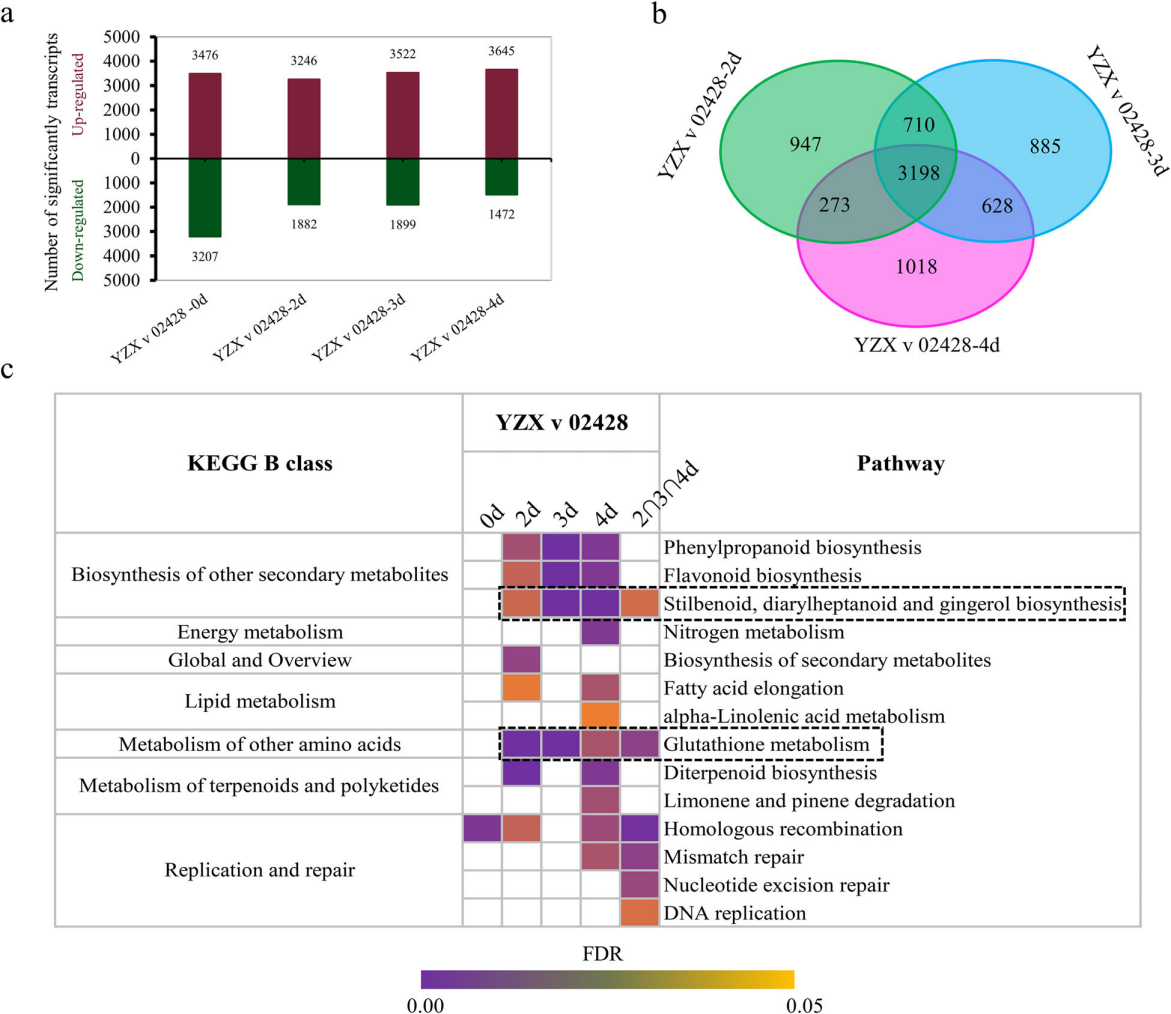
The number of DEGs and the KEGG enrichment analysis of DEGs of 02428 and YZX at days 0, 2, 3, and 4. **a** Up- and downregulated genes detected between 02428 and YZX. **b** Venn diagram of DEGs between 02428 and YZX on days 2, 3 and 4. **c** KEGG enrichment analysis of DEGs of 02428 and YZX at days 0, 2, 3, and 4

In terms of the transcriptome, the largest number of DEGs were detected at day 0, with a total of 6783, and the remaining 3 days had approximately 5000 DEGs. Interestingly, the gene expression level of 02428 was higher than that of YZX at all days (Fig. 6a). Considering that the DEGs at day 0 could not effectively reflect the difference in the germination and young seedling growth of the two materials, we also identified the intersection set of DEGs at days 2, 3, and 4, which contained 3198 DEGs (Fig. 6b). KEGG enrichment analysis was performed for the DEGs at 0, 2, 3, and 4 days and in the intersection set. The results show that 14 pathways were enriched. Notably, glutathione metabolism and phenylpropanoid biosynthesis; flavonoid biosynthesis; and stilbenoid, diarylheptanoid and gingerol biosynthesis were continuously significantly enriched on days 2, 3 and 4. Fortunately, among them, glutathione metabolism and stilbenoid, diarylheptanoid and gingerol biosynthesis were also enriched by DEGs in the intersection set. These results indicated that the four pathways, especially glutathione metabolism and stilbenoid, diarylheptanoid and gingerol biosynthesis, may be involved in mediating the differences in the speeds of 02428 and YZX germination and young seedling growth. With respect to the metabolome, the amount of DEMs increased with time, reaching a maximum of 104 on the fourth day. We also identified the intersection set of DEMs at days 2, 3, and 4, which contained 44 DEMs (Additional file 8: Figure S4a, b). Among them, esculetin O-quinacyl esculetin O-quinic acid, lysoPC 19:0, lysoPC 14:0 (2n isomer), 4-guanidinobutyric acid, and DIMBOA glucoside showed substantial differences (Additional file 8: Figure S4c).

### Expression patterns of genes related to the ascorbate–glutathione (AsA-GSH) pathway

Growing evidence has shown that ROS may function as transmitters or messengers of environmental cues during seed germination and control seed germination [25–29]. The accumulation of ROS during seed germination in different plant species has been reported [19, 30–32]. However, ROS have long been considered hazardous molecules; ROS production by germinating seeds has often been considered a negative effect that might affect the germination process [33]. Therefore, antioxidant mechanisms are considered to be important for successful germination [34]. Within the antioxidant system in plants, the AsA-GSH pathway plays an important and central role in regulating ROS. The KEGG enrichment results suggested that glutathione metabolism pathways were most likely involved in mediating the differences in the speeds of 02428 and YZX germination and young seedling growth. Thus, the AsA-GSH pathway should be an area of focus in the future.

**Fig. 7:**
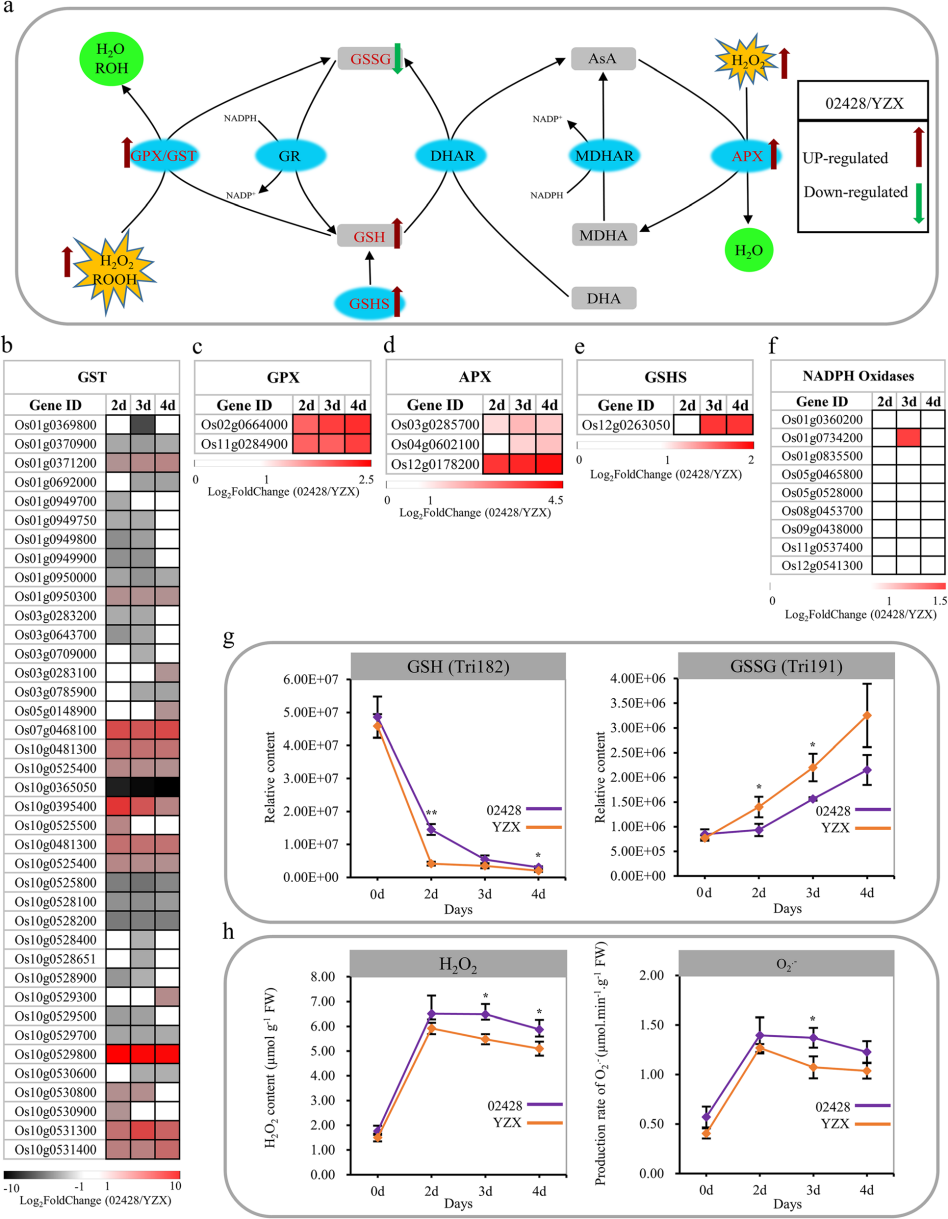
Changes in the AsA-GSH cycle-related genes and metabolites. **a** The AsA-GSH pathway model. Red arrows indicate upregulated expression, and blue indicates downregulated expression. **b**, **c**, **d**, **e** Four types of DEGs were significantly enriched on days 2, 3, and 4 and in the intersection set. **f** Heat map of the fold changes (02428 / YZX) of 9 NADPH oxidase genes in rice. Different colors show the value of the fold change, and blanks indicate that the differential expression of the gene was not significant during this period. **g** Changes in reduced glutathione (GSH) and oxidized glutathione (GSSG) content in 02428 and YZX. **g** Quantification of H_2_O_2_ content and the O_2_^−^ production rate in 02428 and YZX for 0, 2, 3 and 4 days

Under optimal conditions, glutathione is mostly present in the reduced form (GSH). When plants are subjected to suboptimal conditions, oxidized glutathione (GSSG) can accumulate to high levels [35–37]. The maintenance of a high GSH/GSSG ratio is crucial for many physiological functions, and a decrease in this ratio can be used as an indicator of oxidative stress. In this study, both GSH and GSSG were also detected in our metabolite profiles. In particular, the GSH content of both materials continued to decrease, and the GSSG content of both materials continued to increase (Fig. 7g). These results indicated that the two materials were subjected to oxidative stress during germination and young seedling growth. Notably, the contents of GSH and GSSG in YZX changed more rapidly than those in 02428. Therefore, we speculate that the ROS-scavenging ability of YZX is better than that of 02428.

In addition, the expression profiles of DEGs located in the glutathione pathway, which was significantly enriched on days 2, 3, and 4 and in the intersection set were investigated (Fig. 7b, c, d, e). These DEGs mainly contained 2 glutathione peroxidase (GPX) genes, 3 ascorbate peroxidase (APX) genes, 1 glutathione synthetase (GSHS) gene and 39 glutathione *S*-transferase (GST) genes. GPX and APX are two major ROS-scavenging enzymes that catalyze the reduction of H_2_O_2_ to prevent potential H_2_O_2_-derived cellular damage. GSTs also have hydroperoxide activity, but GSTs generally use H_2_O_2_ only at low rates [38]. The overexpression of *GSHS* significantly elevates the GSH content and abiotic stress tolerance [39, 40].

In this study, the expression profiles of GPX, APX and GSHS DEGs showed that the expression levels of these genes were higher in 02428 than in YZX. Although the expression profiles of GSTs had no obvious trend, we still find that among the genes with large multiple differences and a high expression abundance in the two materials, the genes with higher expression levels in 02428 than in YZX account for the majority. This result indicated that the ROS-scavenging system of 02428 is more active than that of YZX on days 2, 3 and 4. Since NADPH oxidases are the major ROS producers in plants [41], we further studied 9 genes of this gene family in rice, and the results showed that these 9 genes, except for Os01g0734200 were not differentially expressed in 02428 and YZX (Fig. 7f). In addition, we also directly detected the H_2_O_2_ content and O_2_^.−^ production rate of the two materials. The results showed that the H_2_O_2_ content and O_2_^.−^ production rate increased from days 0 to 2 and decreased from days 2 to 4 in both 02428 and YZX. Notably, although the H_2_O_2_ content and O_2_^.−^ production rate of 02428 were higher than those of YZX, the differences in these factors between 0 and 2 days were not significant and reached a significant difference during the time of decline from 2 to 4 days, especially for the H_2_O_2_ content (Fig. 7h).

Combined with the above results, we believe that the production and accumulation of ROS mainly occurred in the first 2 days and decreased from days 2 to 4. There is no difference in H_2_O_2_ and O_2_^.−^ production capacity between the two materials. However, YZX had a better ROS-scavenging ability than 02428. Therefore, the higher ROS-scavenging capacity should still be maintained at days 2, 3 and 4 in 02428. Interestingly, this result is also supported by our previous KO enrichment analysis of DEGs in transcriptome expression profiles at different stages of the two materials., i.e., the glutathione metabolism pathway was significantly enriched at three periods in 02428; however, it was significantly enriched at the first two periods in YZX. In summary, the difference in ROS-scavenging abilities mediates the difference in growth speed between 02428 and YZX.

## Discussion

This study conducts a comprehensive profile of the transcriptome and metabolome during germination and young seedling growth in rice. In particular, phenylpropanoid biosynthesis and glutathione metabolism are involved in seed germination and young seedling growth. Howell et al. (2009) studied the changes in embryo transcription and metabolite abundance within 48 h after rice seed imbibition [23]. The largest change in transcript abundances occurred between 3 and 12 h, and there is a relatively small change in transcripts at subsequent time points, in contrast, changes in a large number of metabolites continued up to 48 h. So, they think that the early changes in metabolites arise from the activity of preexisting enzymes, in addition, due to later changes in metabolites occur after changes in transcript abundance, so, later changes in metabolites are likely driven by transcription and translation. In our study, the largest change in transcript abundances occurred between 0 and 2 days, while the change between 2 and 4 days was small. The abundance of metabolites was different from that of transcripts, with the smallest change in metabolite abundances between 0 and 2 days, followed by relatively large changes in metabolites at subsequent time points. Although we investigated metabolites for a long period of time, up to 4 days, the changes in metabolites still lagged behind the changes in transcripts. To further explore the relationship between the metabolites and transcripts, we calculated Pearson correlations in R software for each group. The comparisons revealed a low relevance between the data obtained from the two omics, the absolute value of average correlation coefficient just is 0.34. These results demonstrate that gene expression cannot fully account for the metabolite abundances.

During the germination of rice seeds, a large number of transcripts are involved in cell wall metabolism, lipid metabolism, nucleotide degradation, amino acid synthesis, the TCA cycle, and jasmonate synthesis [23]. These results are almost identical to our results (Fig. 5). MapMan analysis showed that cellulose synthesis, cell wall synthesis, cell wall modification, fatty acid synthesis, tetrapyrrole synthesis, and β-oxidation were upregulated. These observations are further supported by our results (Fig. 4b; Additional file 6). Notably, we also observed several upregulated items, such as light reaction, photorespiration, mitochondrial electron transport, tetrapyrrole synthesis, and amino acid synthesis (Fig. 5). Tetrapyrroles are important to photosynthesis [42], indicating that the seeds have begun to prepare or have already completed seedling establishment and have gradually begun to achieve autotrophy.

By comparing the germination data of rice seeds with those of barley [43] and bread wheat [44], we also found similarities among the three, the upregulation of transcripts encoding components involved in sugar, starch, and lipid metabolism. The seed expands rapidly at the initial stage of germination due to water absorption. Genes associated with cell wall synthesis and degradation were heavily upregulated. This indicates that many fractured cell walls were degraded into small molecules to provide materials and energy for cell wall synthesis during seed germination to further help the establishment of seedling morphology.

A total of 14 continuously DEGs and 15 DEMs in the two materials were identificated. Among the 14 continuously DEGs, there are 6 (Os02g0308800, Os02g0589000, Os03g0382100, Os07g0687100, Os11g0641800, Os12g0515800) have high expression level in the 3-day seedling in the gene atlas of Minghui 63 rice covering the entire life cycle [45]. Which also further proves that our results are reliable. Among the 14 continuously DEMs, there are two flavones, tricin 7-O-hexoside and tricin O-glucuronic acid; the content of tricin 7-O-hexoside in 02428 and YZX continued to increase within 0–4 days. In another study, to investigate the accumulation of flavonoids in different tissues of rice, Dong et al. [46] collected samples from 6 different tissues, including flag leaf, mature culm, panicle at grain filling stage, mature grain, and seed at 72 h after germination and root at vegetative stage. For each tissue, a mixture of samples from 24 rice varieties (12 *indica* and 12 *japonica* rice) were obtained. The results showed that there were 10 flavones, including tricin 7-O-hexoside, displayed higher levels in germinating seed than those in the grain, suggesting the increased amount of flavones synthesis during seed germination. In addition, the accumulation patterns of flavones at different developmental stages were further explored by using the rice varieties of Zhonghua 11 and Zhenshan 97 as materials. The result also showed that tricin 7-O-hexoside increased sharply in the seedlings during the first 10 days after germination, followed by a slight decrease during the later stage [46]. In a word, tricin 7-O-hexoside plays an important role in the germination stage and young seedling growth stage of rice. Auxin plays important roles in the regulation of seed dormancy in Arabidopsis [47]. The tryptamine pathway is one of the pathways for auxin synthesis in plants, and tryptamine to N-hydroxy tryptamine is the rate-limiting step of this pathway [48]. Interestingly, N-hydroxy tryptamine was also included in the 15 continuously DEMs we screened. Taken together, these 14 continuously DEGs and 15 DEGs are ideal candidate biomarkers for rice seed germination and young seedling growth.

The variety 02428 is a typical *japonica* rice variety, while YZX is an *indica* rice variety. The statistically significant differentially expressed transcripts and metabolites in response to germination and young seedling growth were identified. We found that the abundance of both metabolites and transcripts of 02428 was greater than that of YZX. The specific transcripts and metabolites of each material accounted for approximately 20% of the total. Moreover, we also conducted principal component analysis (PCA) of all transcripts and metabolites at different 3 timepoints after imbibition. The results showed that 02428 and YZX were clearly divided into two groups (Additional file 9), and the background of the two materials does have a certain difference. The two materials have a significant difference in germination and young seedling growth speed, with YZX showing a faster speed than 02428 (Fig. 1). Here, to explore the reason for this finding from the perspective of transcription and metabolism, we identified 44 DEMs with significant persistent differences at 2, 3, and 4 days. As for the transcriptome, we conducted KEGG enrichment analysis for the DEGs at days 0, 2, 3, and 4 and in the intersection set. There were four continuously enriched items that were highly important. Of course, the DEGs and DEMs screened by us may be false-positive due to the difference in the material background. However, from another perspective, it is because of the great difference in the material background that we can obtain more, and more valuable target genes and metabolites. This conclusion is backed up by many studies [49**–**52]. As far as our results are concerned, I think the DEMs and DEGs that we have screened for are fairly reliable. Among the 44 DEMs, there were 13 flavonoids metabolites. Flavonoids play an important role in seed germination [46, 53]. As for transcriptome, we identified the intersection set of DEGs at days 2, 3, and 4, which contained 3198 DEGs. Interestingly, in previous studies, we used genetic populations constructed by YZX and 02428 to map QTLs related to seed vigor and obtained a total of 6 important QTLs [54]. Within these 6 QTL intervals, we found a total of 4 genes (Os06g0109600, Os06g0281800, Os06g0282000, Os07g0611600) contained in 3198 DEGs. They are likely the target genes involved in mediating the difference in the germination speed of YZX and 02428. As for the four continuously enriched items, phenylpropanoid biosynthesis and flavonoid biosynthesis were also supported by our MapMan results, that is, the DEGs associated with phenylpropanoid biosynthesis and flavonoid biosynthesis were mostly downregulated in YZX and mostly upregulated in 02428. Flavonoid biosynthesis was also consistent with the dynamic KO enrichment analysis, i.e., flavonoid biosynthesis was enriched only in 02428. Two other items of importance were glutathione metabolism and stilbenoid, diarylheptanoid and gingerol biosynthesis. They were enriched not only at 2, 3 and 4 days but also in the intersection of DEGs at 2, 3 and 4 days. These results were supported by the dynamic KO enrichment analysis, i.e., stilbenoid, diarylheptanoid and gingerol biosynthesis was enriched only in YZX; the glutathione metabolism pathway was significantly enriched at three periods in 02428; however, it was significantly enriched at the first two periods in YZX. Thus, the four pathways were reliable and were involved in mediating the differences in the speeds of 02428 and YZX germination and young seedling growth.

Among these four items, we have taken a further interest in the glutathione metabolism pathway. ROS are unavoidable by-products of aerobic metabolism, whereas, GSH is one of the most important cellular antioxidants since it can scavenge ROS directly or indirectly [55]. In this study, the change in the GSH/GSSG ratio indicated that both materials were subjected to oxidative stress. We further studied 9 genes of this gene family in rice and directly detected the H_2_O_2_ content and O_2_^.−^ production rate of the two materials. The results showed that there was no significant difference in the production capacity of ROS between the two materials. However, the changes in the H_2_O_2_ content and O_2_^.−^ production rate from 2 to 4 days indicated that the ROS in YZX were cleared faster than the ROS in 02428. We further speculated that this difference was mainly caused by the ROS-scavenging mechanism. Therefore, we analyzed the genes enriched in the AsA-GSH pathway and differentially expressed in the two materials. Among these genes, GPX DEGs, APX DEGs, and GSHS DEGs had higher expression levels in 02428 than in YZX. Among the 39 GST genes with large multiple differences and high expression abundance in the two materials, the genes with increased expression levels in 02428 accounted for the majority. We further validated the expression levels of two GPX genes (Os02g0664000 and Os11g0284900), three APX genes (Os03g0285700, Os04g0602100 and Os12g0178200), and one GSHS gene (Os12g0263050) using the differences in qRT-PCR. Among the 39 GST DEGs, two genes (Os10g0529800 and Os10g0525800) with multiple large differences and an increased expression abundance in the two materials were also chosen. The results of this analysis demonstrated that both methods generated similar results; Pearson correlation coefficients (r) for these eight genes ranged between 0.80 and 0.96 (Additional file 10). Specifically, we believe that these genes lead to the difference in ROS-scavenging abilities between the two materials, which in turn affects the difference in germination and growth speed of the two materials. In addition, to better understand the relationship between metabolites and genes in AsA-GSH pathway. We screened a total of 200 DEGs with absolute values of correlation coefficients of GSH and GSSG greater than or equal to 0.70 from 3198 DEGs. A network based on Pearson correlation coefficient was established and visualized using Cytoscape software (version 3.6.1) (Only correlation pairs with an absolute value of correlation coefficient ≥ 0.9 and a *p* value ≤0.05 were included in the analysis). Notably, 1 APX gene, 1 GPX gene, and 2 GST genes are contained in our network (Additional file 11).

## Conclusions

We investigated for the first time the genome-wide expression profile and metabolism profile of all tissues in an *indica* rice and a *japonica* rice during germination and young seedling growth. We identified statistically significant differentially expressed transcripts and metabolites in response to germination and young seedling growth. Among them, we identified 14 shared transcripts and 15 shared metabolites that were continuously differentially expressed in both materials, which may be essential for seed germination and early seedling growth. Enrichment analysis of the DEGs in the transcriptome expression profiles at different stages revealed that phenylpropanoid biosynthesis and glutathione metabolism were continuously enriched, indicating that they were continuously involved in seed germination and young seedling growth. Next, the differences in germination and young seedling growth of the two materials were explored from the transcriptome and metabolome levels. KO enrichment analysis was conducted by using the DEGs of the two materials at 2, 3 and 4 days, and 14 pathways were enriched. Additionally, 44 metabolites with differential expression at 2, 3 and 4 days were identified. Further attention was focused on the AsA-GSH pathway, and it was found that differences in ROS-scavenging abilities mediated by some APX, GPX and GST genes may be involved in mediating differences in the germination and young seedling growth speed of the two materials.

## Methods

### Plant materials and treatment conditions

Two types of rice seeds were used in this study: the *indica* variety YZX and the *japonica* variety 02428. YZX and 02428 were bred by Hunan Academy of Agricultural Sciences and Jiangsu Academy of Agricultural Sciences, respectively. They were grown in a paddy field at the South China Agricultural University, Guangzhou, China (at approximately 113°E longitude and approximately 23°N latitude), in the dry season in 2017. Considering that seed maturity affects germination, seeds were harvested on the 40th day after heading.

The seeds were placed in a 50 °C oven and dry-heat treated for 7 days to break dormancy. Then, the seeds were manually dehulled, surface sterilized with 20% diluted bleach (6–7% NaClO) for 20 min and then rinsed three times with sterile distilled water. Thirty seeds were placed in a petri dish and covered with two layers of 9 cm circular pieces of filter paper, and 10 ml sterilized distilled water was added. Each sample was repeated three times. All petri dishes were placed in a chamber with an 8 h light (200 μmol m^− 2^ s^− 1^)/16 h dark cycle at 30 °C. All tissues (seed + seedling) of germinated YZX and 02428 seeds were collected on days 0, 2, 3, and 4 and stored at − 80 °C prior to RNA and metabolite extraction. And 0–2 days is the germination stage, 2–4 days is young seedling growth stage.

Five plants were randomly selected from each petri dish to measure the bud length with a standard ruler.

### RNA-Seq and data analysis

Total RNA from each sample was homogenized in liquid nitrogen using a mortar and pestle and then purified using the Plant Total RNA Purification Kit (ComWin Biotech Company) following the manufacturer’s instructions. RNA-Seq library construction and sequencing was performed according to a previous protocol [56]. Quality control was confirmed with Illumina HiSeq software, and all reads passing the filtering specifications were mapped onto the reference genome IRGSP-1.0.

After the expression level of each transcript and gene was calculated, differential expression analysis was conducted using edgeR [57]. The false discovery rate (FDR) was used to determine the *P* value threshold in multiple tests, and for the analysis, a threshold of FDR ≤ 0.05 and an absolute value of a fold change ≥ 2.0 were used to assess the significance of gene expression. Kyoto Encyclopedia of Genes and Genomes (KEGG) and Gene Ontology (GO) pathway enrichment analysis were performed using the OmicShare tools (www.omicshare.com/tools); the groups with FDR ≤ 0.05 were considered significantly enriched.

MapMan software (ver. 3.6.0RC1) [24] was used to map transcriptome data, define functional categories, and perform time course analyses for identifying significantly overrepresented functional groups. Osa_RAPDB_v1 mapping files were downloaded from the MapManStore server (http://mapman.gabipd.org/web/guest/mapmanstore).

### Metabolomic analysis

Metabolite profiling was carried out using a widely targeted metabolome method by Wuhan Metware Biotechnology Co., Ltd. (Wuhan, China) (http://www.metware.cn/). Reagents and methods for extracting metabolites were all carried out as outlined by Yan et al. [58]. All sample extracts were analyzed using a liquid chromatography electrospray ionization tandem mass spectrometry (LC-ESI-MS/MS) system (HPLC, Shim-pack UFLC SHIMADZU CBM30A system, www.shimadzu.com.cn/; MS, Applied Biosystems 6500 Q TRAP, www.appliedbiosystems.com.cn/). The analytical conditions were as follows. HPLC column, Waters ACQUITY UPLC HSS T3 C18 (1.8 μm, 2.1 mm*100 mm); solvent system, water (0.04% acetic acid): acetonitrile (0.04% acetic acid); gradient programme, 100:0 (V/V) at 0 min, 5:95 (V/V) at 11.0 min, 5:95 (V/V) at 12.0 min, 95:5 (V/V) at 12.1 min, and 95:5 (V/V) at 15.0 min; flow rate, 0.40 ml/min; temperature, 40 °C; and injection volume, 2 μl. The effluent was alternatively connected to an ESI-triple quadrupole-linear ion trap (Q TRAP) mass spectrometer.

Linear ion trap (LIT) and triple quadrupole (QQQ) scans were acquired with a Q TRAP mass spectrometer, API 6500 Q TRAP LC/MS/MS System, equipped with an ESI Turbo Ion-Spray interface, operating in the positive ion mode and controlled by Analyst 1.6 software (AB Sciex, Framingham, MA, USA). The ESI source operation parameters were as follows: ion source, turbo spray; source temperature, 500 °C; ion-spray voltage (IS), 5500 V; ion source gas I (GSI), gas II (GSII), and curtain gas (CUR) of 55, 60, and 25 psi, respectively; and high collision gas (CAD). Instrument tuning and mass calibration were performed with 10 and 100 μmol/L polypropylene glycol solutions in QQQ and LIT modes, respectively. The QQQ scans were acquired as MRM experiments with collision gas (nitrogen) set to 5 psi. Declustering potential (DP) and collision energy (CE) for individual MRM transitions was performed with further DP and CE optimization [59]. A specific set of MRM transitions was monitored for each period according to the metabolites eluted within this period. Qualitative and quantitative determination of metabolites followed the methods of Li et al. [60].

The significant differences in the relative metabolite content was tested by orthogonal partial least squares discriminant analysis (OPLS-DA). For the analysis, a threshold of variable important in projection (VIP) value ≥1 and univariate statistical analysis of the test value *P* value > 0.05 were used. OPLS-DA was carried out by using R software version 3.5.0 (http://www.r-project.org/).

### Quantification of ROS

To quantify O_2_^·–^, the production rate (nmol O_2_^·–^ min^− 1^ g FW^− 1^) was analyzed as described in Zhang et al. [61]. For this quantification, all tissues from 5 seeds at the indicated imbibition time points were extracted in 2 ml of 50 mM phosphate buffer containing 1 mM EDTA, 0.3% Triton X-100, and 2% PVP (pH 7.8). The homogenate was centrifuged at 12, 000 rpm for 20 min. A 1 ml aliquot of supernatant solution was mixed with 1 ml of 50 mM phosphate buffer (pH 7.8) and 1 ml of 1 mM hydroxylamine hydrochloride and incubated at 25 °C for 1 h. After the addition of 1 ml of 17 mM *p*-amino benzene sulfonic acid and 1 ml of 7 mM α-naphthylamine, the mixture was incubated at 25 °C for 20 min. The absorbance of the obtained solution was read at 530 nm. A standard response curve was prepared with a known concentration of NO_2_^−^ using the same method as that described above.

H_2_O_2_ was extracted by the homogenization of all tissues from 5 seeds from each of the indicated time points in 3 mL of cold acetone. The homogenate was centrifuged at 12,000 rpm for 15 min. A 1 mL aliquot of supernatant solution was mixed with 0.1 mL of 5% titanium sulfate in concentrated HCl, and then, 0.2 mL aqueous NH_3_ (25%) was added to precipitate the peroxide–titanium complex. The mixture was then centrifuged at 12,000 rpm for 15 min. The precipitate was solubilized in 3 mL of 2 mM H_2_SO_4_. Absorbance was then read at 415 nm. A standard response curve was prepared with a known concentration of H_2_O_2_ using the same method as that described above. Mean values ±SD of three biological replicates were calculated.

### Validation of candidate genes by real-time quantitative RT-PCR

All the methods used were described in our previous report [54]. RNA samples were reverse transcribed into cDNA using the high-capacity cDNA archive kit (Applied Biosystems, USA). qRT-PCR was conducted using the AceQ qPCR SYBR Green Master Mix Kit (Vazyme Biotech) according to standard protocol, and the expression levels of the genes were determined on a StepOnePlus System (Applied Biosystems, USA). Three replicates were used for each treatment. As an endogenous control, Actin was used to normalize the obtained Ct values, and the relative expression values were calculated by the ΔΔCt method. Gene-specific primers were designed using NCBI primer BLAST (http://www.ncbi.nlm.nih.gov/tools/primer-blast/). The primer sequences of the twelve candidate genes are listed in Additional file 12.

## Supplementary information

**Additional file 1: Table S1.** Summary of read mapping statistics.

**Additional file 2: Table S2.** Gene expression data and annotations. All DEGs of 02428. All DEGs of YZX. Intersection of 02428 and YZX.

**Additional file 3: Table S3.** Detected metabolites and relative content.

**Additional file 4: Figure S1.** GO and KEGG analyses for the core DEGs. (a) Significant cellular component GO terms. (b) Significant biological process GO terms. (c) Significant molecular function GO terms. (d) Significant KEGG pathways.

**Additional file 5: Table S4.** Identification of continuously differentially expressed genes and metabolites.

**Additional file 6: Figure S2.** MapMan metabolism overview maps showing differences in transcript levels during seed germination and young seedling growth of YZX. (a) 0 days v 2 days. (b) 2 days v 3 days. Log2 ratios for average transcript abundance were based on three replicates. The log_2_-fold change color scale ranges from − 3.5 to 3.5, where blue represents downregulated transcripts, and red represents upregulated transcripts.

**Additional file 7: Figure S3.** GO analyses for the DEGs (0 v 2 days, 2 v 3 days, 3 v 4 days) in 02428 and YZX. Different colors show the values of FDR, and blanks indicate items that were not significantly enriched during this period.

**Additional file 8: Figure S4.** The number of DEMs at days 0, 2, 3, and 4 of 02428 and YZX. (a) Up- and downregulated genes detected between 02428 and YZX. (b) Venn diagram of DEGs between 02428 and YZX on days 2, 3 and 4. (c) Heat map of the fold changes in the 44 metabolites in the intersection.

**Additional file 9: Figure S5.** PCA results. (a) Transcriptome; (b) metabolome.

**Additional file 10: Figure S6.** qRT-PCR validation.

**Additional file 11: Figure S7.** Co-expression analysis of metabolites and genes in AsA-GSH pathway. Red edges represent positive correlations and blue edges represent negative correlations.

**Additional file 12: Table S5.** Primers used for qRT-PCR.

## Data Availability

All the sequence data generated in this research was deposited in the Sequence Read Archive database (www.ncbi.nlm.nih.gov/sra) at NCBI (National Center for Biotechnology Information) under accession number: SRP277875. The data sets supporting the results of this study are included in the manuscript. Rice seeds are available from the National Engineering Research Center of Plant Space Breeding, PR China.
