# Supplementary figures and images for: Dynamic transcriptome and metabolome analyses of two types of rice during the seed germination and young seedling growth stages
Source: BMC Genomics. 2020 Aug 31;21:603. doi: 10.1186/s12864-020-07024-9 (PMC7460786; doi:10.1186/s12864-020-07024-9)

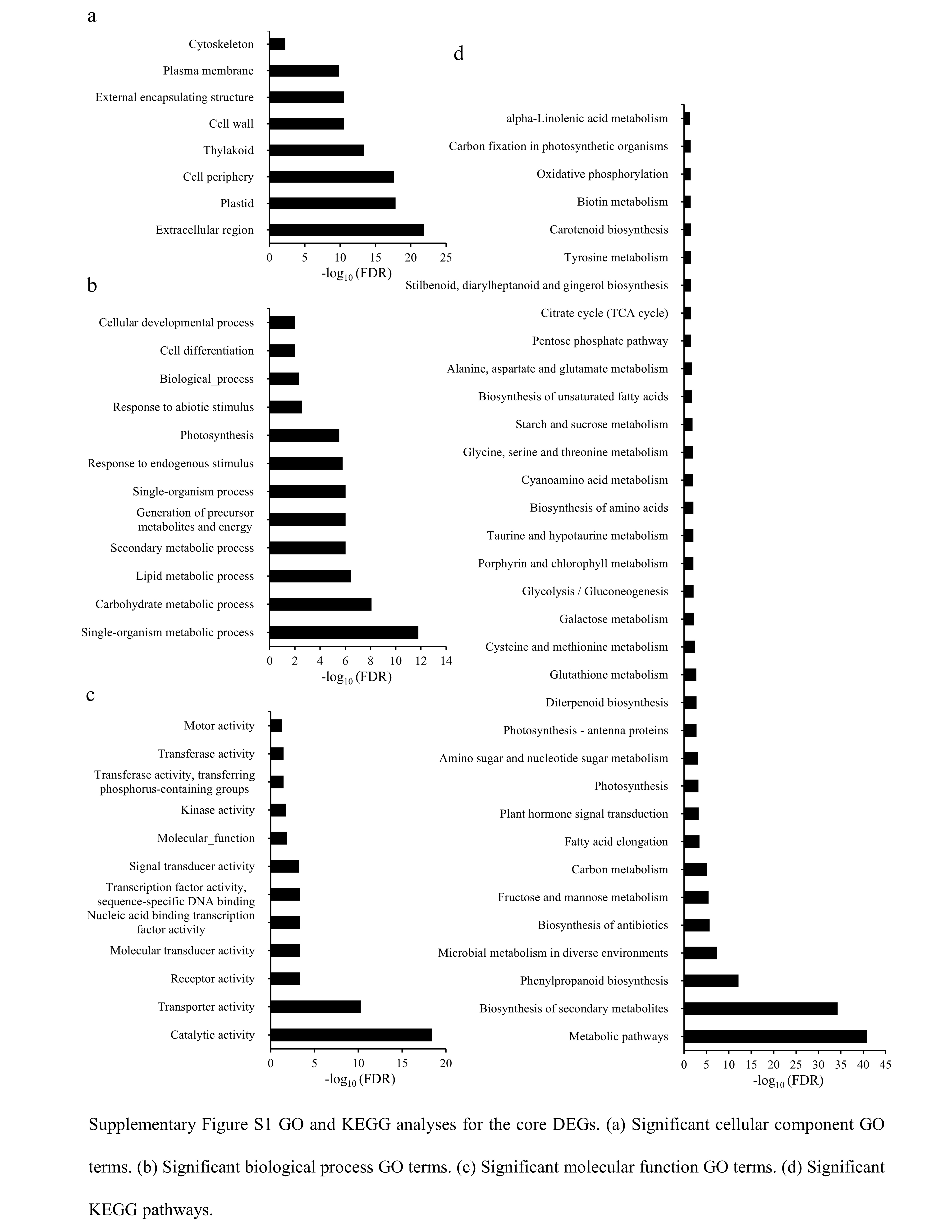

Supplement: Supplementary file 4 — Additional file 4: Figure S1. GO and KEGG analyses for the core DEGs. (a) Significant cellular component GO terms. (b) Significant biological process GO terms. (c) Significant molecular function GO terms. (d) Significant KEGG pathways. [file 12864_2020_7024_MOESM4_ESM.tif]

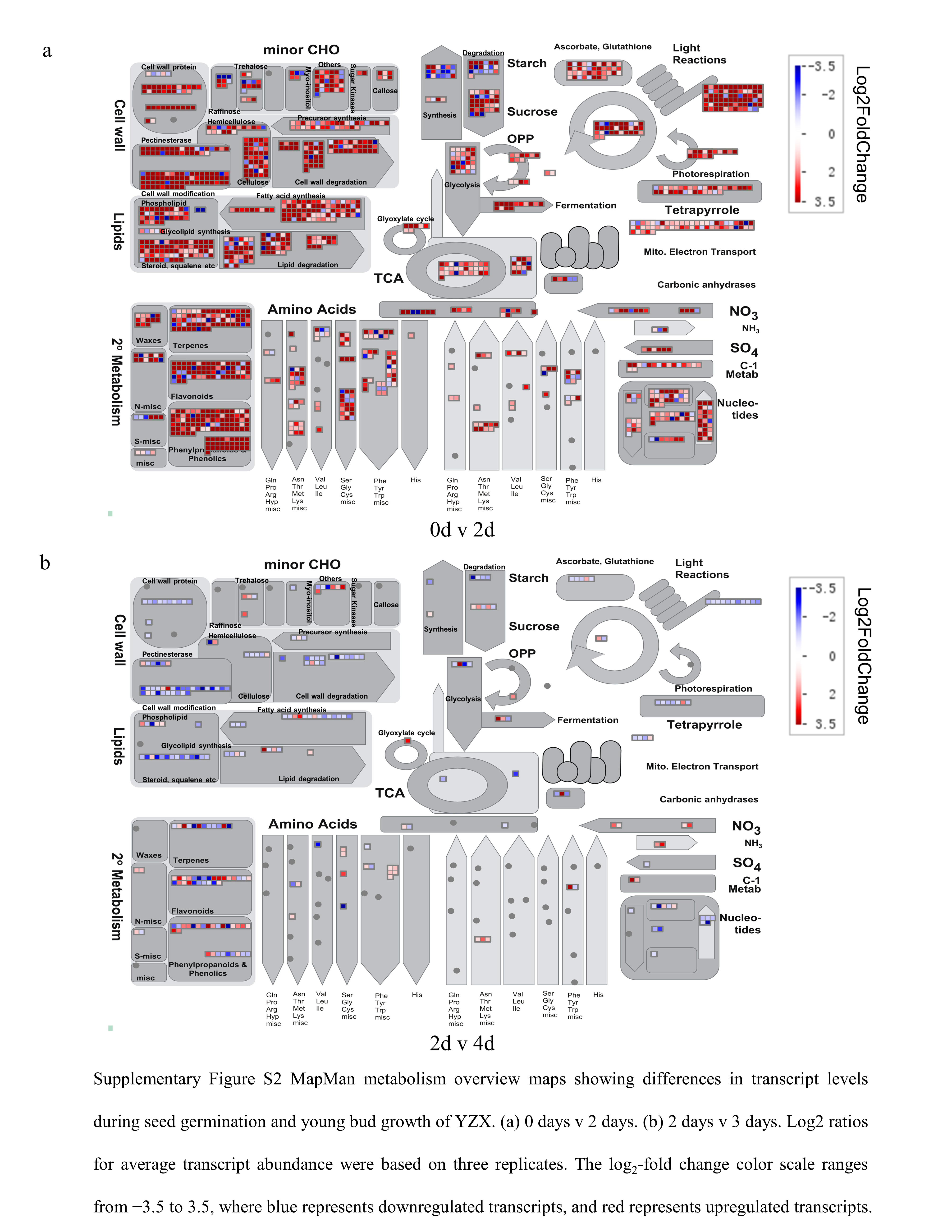

Supplement: Supplementary file 6 — Additional file 6: Figure S2. MapMan metabolism overview maps showing differences in transcript levels during seed germination and young seedling growth of YZX. (a) 0 days v 2 days. (b) 2 days v 3 days. Log2 ratios for average transcript abundance were based on three replicates. The log2-fold change color scale ranges from − 3.5 to 3.5, where blue represents downregulated transcripts, and red represents upregulated transcripts. [file 12864_2020_7024_MOESM6_ESM.tif]

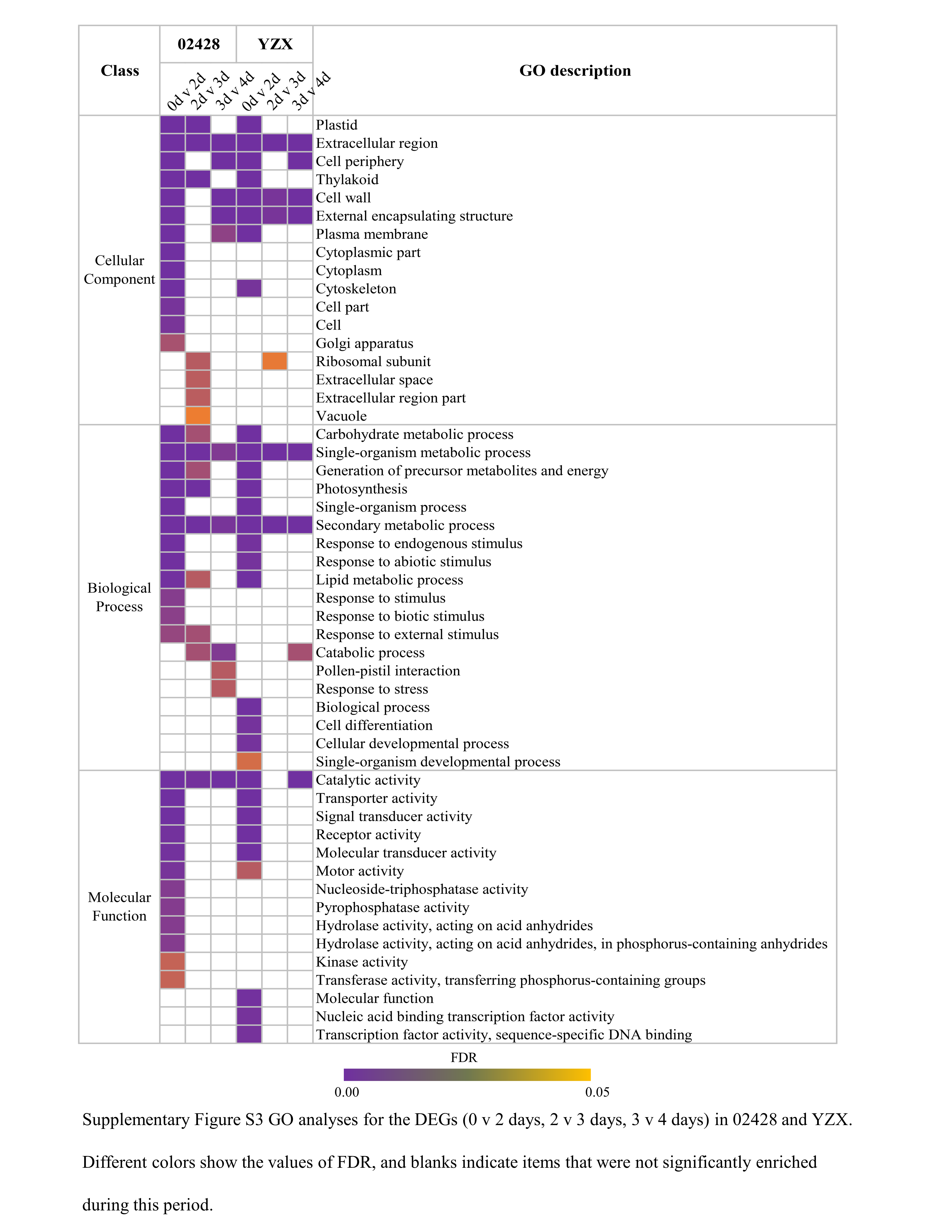

Supplement: Supplementary file 7 — Additional file 7: Figure S3. GO analyses for the DEGs (0 v 2 days, 2 v 3 days, 3 v 4 days) in 02428 and YZX. Different colors show the values of FDR, and blanks indicate items that were not significantly enriched during this period. [file 12864_2020_7024_MOESM7_ESM.tif]

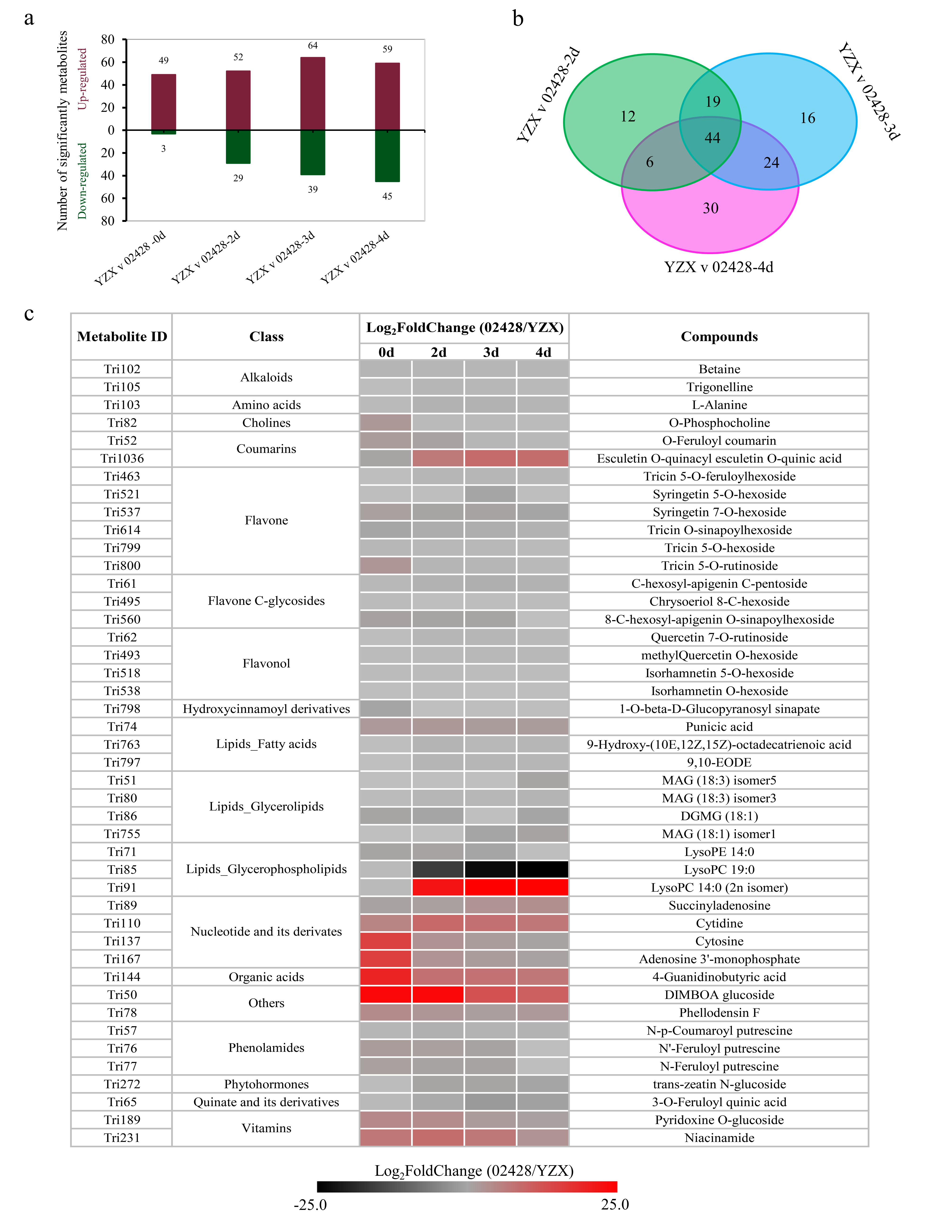

Supplement: Supplementary file 8 — Additional file 8: Figure S4. The number of DEMs at days 0, 2, 3, and 4 of 02428 and YZX. (a) Up- and downregulated genes detected between 02428 and YZX. (b) Venn diagram of DEGs between 02428 and YZX on days 2, 3 and 4. (c) Heat map of the fold changes in the 44 metabolites in the intersection. [file 12864_2020_7024_MOESM8_ESM.tif]

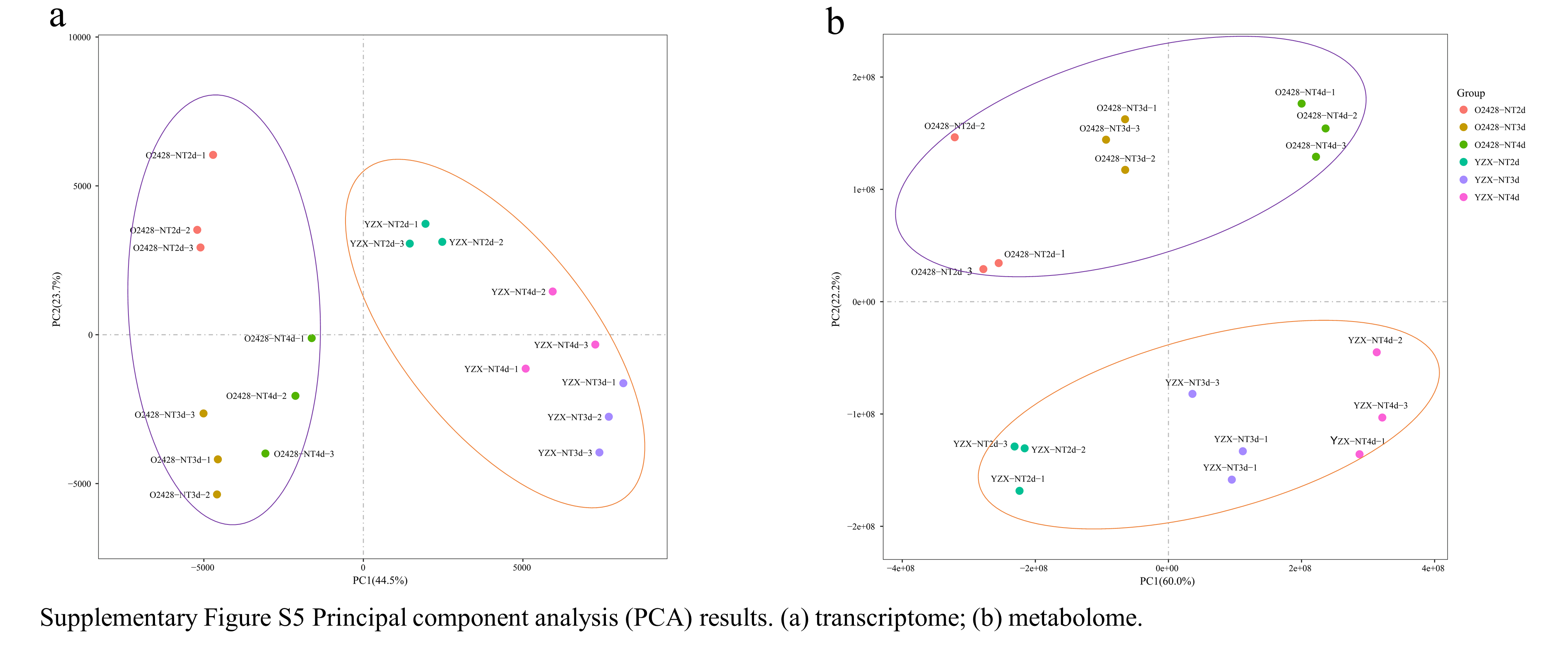

Supplement: Supplementary file 9 — Additional file 9: Figure S5. PCA results. (a) Transcriptome; (b) metabolome. [file 12864_2020_7024_MOESM9_ESM.tif]

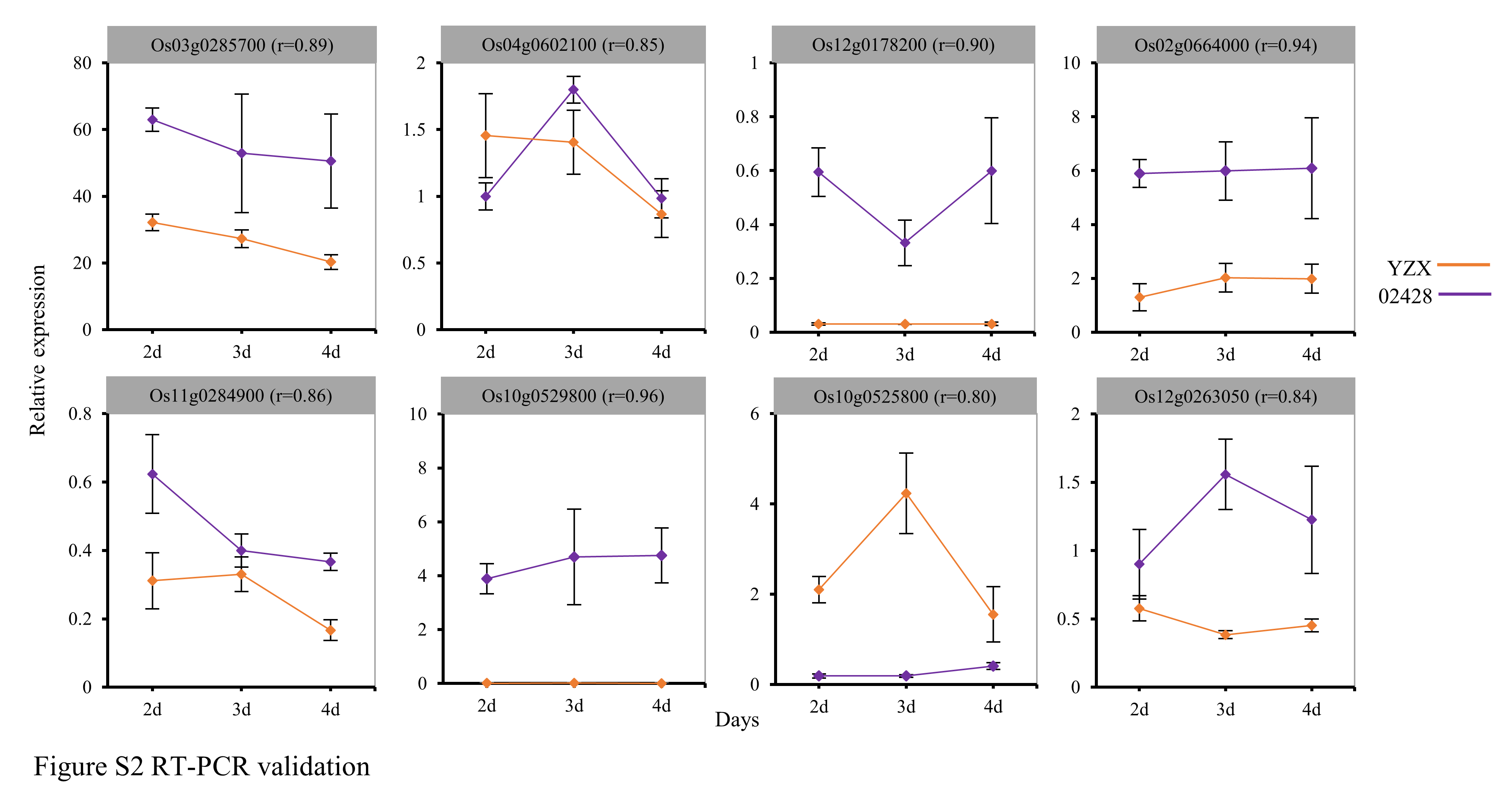

Supplement: Supplementary file 10 — Additional file 10: Figure S6. qRT-PCR validation. [file 12864_2020_7024_MOESM10_ESM.tif]

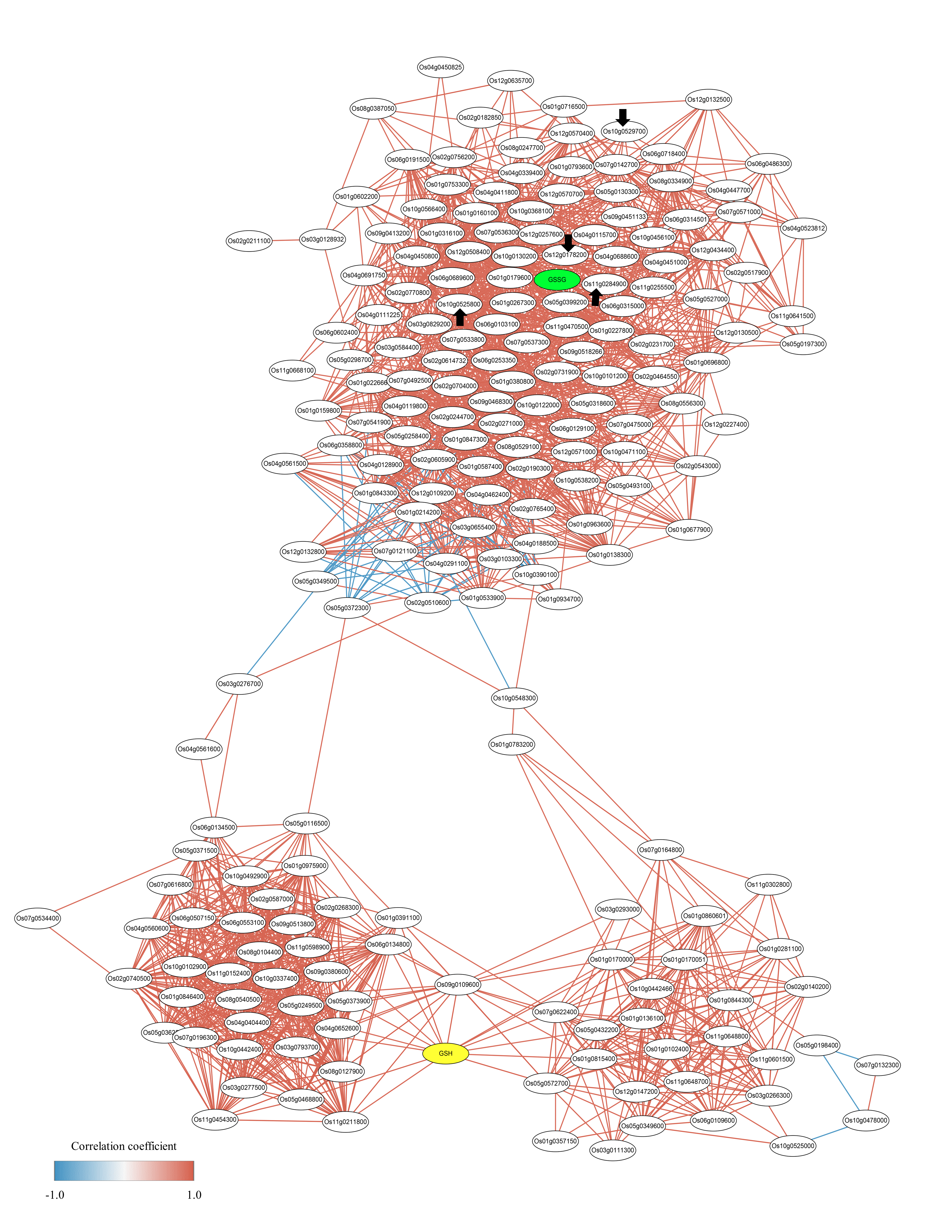

Supplement: Supplementary file 11 — Additional file 11: Figure S7. Co-expression analysis of metabolites and genes in AsA-GSH pathway. Red edges represent positive correlations and blue edges represent negative correlations. [file 12864_2020_7024_MOESM11_ESM.tif]
